# Rapid Detection and Subtyping of Human Influenza A Viruses and Reassortants by Pyrosequencing

**DOI:** 10.1371/journal.pone.0023400

**Published:** 2011-08-19

**Authors:** Yi-Mo Deng, Natalie Caldwell, Ian G. Barr

**Affiliations:** 1 WHO Collaborating Centre for Reference and Research on Influenza, Victorian Infectious Disease Reference Laboratory (VIDRL), Melbourne, Victoria, Australia; 2 School of Applied Sciences, Monash University, Churchill, Victoria, Australia; Kantonal Hospital St. Gallen, Switzerland

## Abstract

**Background:**

Given the continuing co-circulation of the 2009 H1N1 pandemic influenza A viruses with seasonal H3N2 viruses, rapid and reliable detection of newly emerging influenza reassortant viruses is important to enhance our influenza surveillance.

**Methodology/Principal Findings:**

A novel pyrosequencing assay was developed for the rapid identification and subtyping of potential human influenza A virus reassortants based on all eight gene segments of the virus. Except for HA and NA genes, one universal set of primers was used to amplify and subtype each of the six internal genes. With this method, all eight gene segments of 57 laboratory isolates and 17 original specimens of seasonal H1N1, H3N2 and 2009 H1N1 pandemic viruses were correctly matched with their corresponding subtypes. In addition, this method was shown to be capable of detecting reassortant viruses by correctly identifying the source of all 8 gene segments from three vaccine production reassortant viruses and three H1N2 viruses.

**Conclusions/Significance:**

In summary, this pyrosequencing assay is a sensitive and specific procedure for screening large numbers of viruses for reassortment events amongst the commonly circulating human influenza A viruses, which is more rapid and cheaper than using conventional sequencing approaches.

## Introduction

Influenza A viruses contain 8 negative strand RNA segments encoding 11 proteins including polymerase basic protein 2 (PB2) and 1 (PB1), PB1-F2, polymerase acidic protein (PA), hemagglutinin (HA), nucleoprotein (NP), neuraminidase (NA), matrix protein 1 and 2 (M1 and M2), and non-structural protein 1 and 2 (NS1 and NS2) [1]. When different subtypes of influenza A viruses co-infect a cell, they can exchange their gene segments and produce novel viruses with new genotypes, a process known as reassortment. Reassortment plays a pivotal role in the evolution of influenza viruses, and the recent 2009 H1N1 pandemic virus (H1N1pdm) was the result of multiple reassortment events between human, avian and swine influenza A viruses [2], [3].

Since the outbreak of the 2009 pandemic, H1N1pdm has quickly become a dominant influenza strain circulating throughout the world, although seasonal H3N2, as well as influenza B viruses, continue to co-circulate.

Co-infection of H1N1pdm with seasonal H1N1 or H3N2 viruses was sometimes detected in patients [4], and hence it is possible that reassortants might arise from these viruses or other animal influenza viruses. Indeed, there have been several reported cases so far where the H1N1pdm subtype was reassorted with swine influenza viruses [5], [6], [7]. Fortunately, these reassortants were self-limited and did not spread further into the wider population. Nevertheless, these cases highlight the urgent need for the development of a rapid and robust method that allows any further reassortment of the H1N1pdm virus to be quickly identified before it can spread further. At present, influenza virus reassortants can be detected by full-genome sequencing, although this is accurate, it is a time-consuming and labor-intensive technique that is not able to be done in many laboratories. Recently, real-time RT-PCR methods have also been developed to detect known reassortants between H1N1pdm and seasonal H1N1 viruses [4], [8], [9], however, these assays are limited in that no sequence data could be generated.

Pyrosequencing is a relatively new technology that is based on the principle of sequencing by synthesis [10]. It has the capacity of generating accurate sequence information for at least 50 nucleotides in real-time, and the capacity of high throughput screening of up to 96 samples within one hour. Combining the benefits of both sequencing and real-time PCR, pyrosequencing has been widely used in influenza molecular surveillance for SNP detection and mutation screening, such as antiviral resistant mutations [11], [12], [13], [14], [15]. However, these methods are designed to only detect point mutations within its short target region. Here we report for the first time the development of a pyrosequencing-based assay that is capable of not only subtyping human influenza A viruses but also detecting potential new reassortants among H1N1pdm, seasonal H1N1 and H3N2 based on signature sequences (15–30 bp) from all eight gene segments.

## Results

### Pyrosequencing assay development

**Figure 1:**
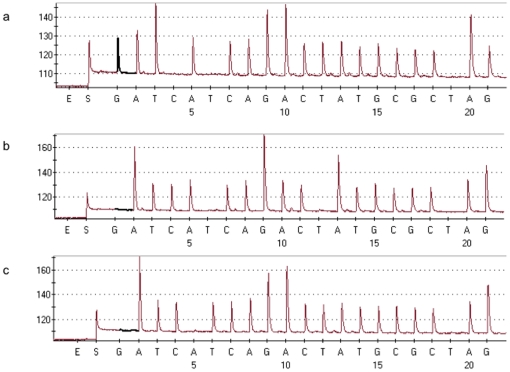
Pyrograms of PA gene segment of H1N1pdm, H3N2, and seasonal H1N1 viruses. The *y* axis represents the luminescence intensity, of which the peak heights are proportional to the number of each nucleotide incorporated at one time. The order of enzyme (E), substrate (S), and sequential nucleotide addition is indicated on the *x* axis. (a) A/Auckland/1/2009 (H1N1pdm) sequence GATTACAGGAACTATGCGCAAG. (b) A/Brisbane/10/2007 (H3N2) sequence AATCACAGGACAATGCGCAGG. (c) A/Brisbane/59/2007 (seasonal H1N1) sequence AATCTCAGGAACTATGCGCAGG.

The nucleotide sequences of selected viruses from the influenza A subtypes H1N1, H3N2, and H1N1pdm were downloaded from NCBI influenza database (http://www.ncbi.nlm.nih.gov/genomes/FLU/Database). In general, all internal genes (PB2, PB1, PA, NS, NP and M) across the three subtypes shared high sequence homology, therefore the same PCR and sequencing primers were designed to target the conserved region of all three subtypes to minimize the number of primer sets used in this assay. Due to the high sequence variation within the HA and NA regions from the H1N1 and H3N2 subtypes, two pairs of primers were designed, one specific for H3N2, the other targeting both H1N1pdm and seasonal H1N1 (except for the N1 forward primer). Then a sequencing primer was designed which targeted the conserved regions of all viruses immediately adjacent to a region that was diverse between different subtypes but highly conserved within the same subtypes. When pyrosequencing was performed with these sequencing primers using a pre-designed sequencing order, different subtypes of influenza A could be distinguished with the short signature sequences (varied from 15 to 30 bp) unique to each of the genes. A set of representative pyrograms is shown in Figure 1, clearly depicting three different signature sequences of the PA gene segments for H1N1pdm, H3N2 and the seasonal H1N1. The sequences obtained were then analysed using the Identifier program and compared with the reference sequences in a purpose-built library containing the signature sequences of each gene from all subtypes. Automatic detection of subtype and gene segment was generated for each sequence, and a score was also given to indicate how strong the homology was to the reference sequence. To gain as much information as possible, the regions to be pyrosequenced also covered different gene regions of interest; for example, the HA sequence of H3N2 could be further differentiated between the two lineages (A/Brisbane/10/2007 and A/Perth/16/2009), and the NA sequences to be distinguished from the wild-type oseltamivir-sensitive H275 and oseltamivir-resistant H275Y mutation, and the M2 sequences to be differentiated from adamantane sensitive S31 and the N31 adamantane resistant viruses.

The pyrosequencing assay was first evaluated on 57 viruses isolated from MDCK virus isolates which were typed/subtyped by real-time RT-PCR. This included 13 seasonal H1N1, 16 H3N2, 28 H1N1pdm viruses sampled from 2007 to 2010. All 57 viruses were identified correctly for their subtypes and/or clades for all 8 genes. Moreover the data also provided further information on the NA and MP genes to identify whether they had acquired the H275Y oseltamivir-resistance mutation in the NA or the S31N adamantane-resistance mutation in the M2 protein. All of the eight genes from all 49 viruses were from the currently circulating subtypes, ie H1N1pdm, H3N2 or seasonal H1N1. No reassortant virus was detected. Further conventional full genome sequencing of eight viruses (4 H1N1pdm, 2 seasonal H1N1, 2 H3N2) confirmed the pyrosequencing results.

The specificity of this pyrosequencing method was tested on a number influenza B viruses, and none of them could be detected by any of the 8 gene segments (data not shown), demonstrating that the assay was highly specific to influenza A viruses.

### Subtyping of influenza viruses from original specimens

To further test the usefulness of this assay on original specimens, 31 human influenza A samples with Ct values from 20–30 for influenza A M gene were chosen for pyrosequencing, including 23 pdmH1N1 and 8 H3N2. Seventeen of them (54%) were correctly subtyped for all 8 genes, with a median Ct value of 22.5 (range 20–27); whereas 14 samples with a median Ct value of 28.5 (range 28–30) could only be partially subtyped by 2–7 genes, but the subtyping results obtained were consistent with the real-time RT-PCR results. This association with Ct value suggested that the lack of pyrosequencing signals for some of the samples was most likely caused by the lower viral loads in these samples.

### Identification of reassortant viruses

The ability of this assay to identify any reassortant virus with mixed genes from different subtypes was tested on 5 viruses, including the vaccine production reassortant viruses IVR-148 (A/Brisbane/59/2007), IVR-153 (A/California/07/2009) and IVR-157 (A/Victoria/208/2009), and three H1N2 viruses (A/Bangkok/335/2000, A/Singapore/44/2000 and A/Singapore/63/2001). All genes were correctly classified into their corresponding subtypes for these viruses (Table 1), indicating the robustness of this pyrosequencing method to identify any potential reassortant virus.

**Table 1:** Pyrosequencing result for reassortant viruses.

Virus	Gene Subtype^1^							
	HA	NA	PB2	PB1	PA	MP	NP	NS
**IVR-148**	H1N1	H1N1	PR8	H3N2	PR8	PR8	PR8	PR8
**IVR-157**	H3N2	H3N2	PR8	PR8	PR8	PR8	PR8	H3N2
**IVR-153**	H1N1pdm	H1N1pdm	PR8	H3N2	PR8	PR8	PR8	PR8
**A/Bangkok/335/2000**	H1N1	H3N2	H3N2	H3N2	H3N2	H3N2	H3N2	H3N2
**A/Singapore/44/2000**	H1N1	H3N2	H3N2	H3N2	H3N2	H3N2	H3N2	H3N2
**A/Singapore/63/2001**	H1N1	H3N2	H3N2	H3N2	H3N2	H3N2	H3N2	H3N2

1 Subtype of each gene was determined by automatic comparison to the purpose-built library using Identifier. Signature sequences of the consensus sequences for each gene from H1N1, H3N2, pdmH1N1, as well as the sequence from A/Puerto Rico/8/1934 (PR8) are included in the library.

### Identification of influenza A viruses with unknown subtypes

Six original human influenza A clinical samples whose subtypes could not be identified previously by real-time RT-PCR were selected for pyrosequencing assay in an attempt to determine their subtypes. The results showed that two of the samples with Ct values of 27 and 29 were identified as H1N1pdm for all eight genes, whereas the other four samples with a Ct value between 30 to 33 failed to generate PCR products and subsequent sequencing data. Although the primers were designed to detect human influenza A viruses, some internal primers, such as MP and NS could theoretically also work on most of avian influenza A viruses based on bioinformatics analysis (data not shown). Therefore, the negative results were most likely caused by the low viral loads of these samples.

## Discussion

The 2009 influenza A H1N1 pandemic viruses are continuously evolving, and there is a concern that they may acquire gene segments through reassortment with other circulating viruses from both human and animal sources to produce a new virus which could have higher pathogenicity in man. The H1N1pdm virus is already a product of multiple reassortment events [2], and it is possible that it could reassort with the currently circulating H3N2 virus to generate H1N2 viruses as happened with seasonal H1N1 and H3N2 viruses in early 2000 [16], [17] or viruses with reassorted internal genes.

It has been a challenge to detect reassortant viruses in a quick and robust way. There are a number of methods that have been used for reassortment detection, including real-time RT-PCR [4], [8], [9], conventional RT-PCR and conventional sequencing. The pyrosequencing assay reported here combines the benefits of some of these methods as it is a fast and high-throughput method that only takes about 4 hours to detect all eight gene segments of up to 12 viruses. Furthermore, the method is very accurate and sensitive, and can be used for both original clinical specimens (providing there are sufficient viruses in the sample) and cell or egg grown viruses. Most importantly, with a total of 10 sets of primers, all eight genes of H1N1pdm, seasonal H1N1 and H3N2 or any new reassortant viruses could be accurately subtyped by short sequences without prior knowledge of the virus genome. Clearly, this is not possible for real-time RT-PCR, since specific probes are needed for each gene from each subtype to determine the subtypes of each gene segments. Hence, the pyrosequencing method can greatly reduce the workload and cost of testing for reassrotment. However, the requirement of the PyroMark instrument for this assay might limit its use in some developing countries, as this device is currently not as widely used as conventional sequencers which are available in many laboratories. Another limitation of the assay is that it can only accurately sequence up to 50 bases, so is not suitable for full genome sequencing.

Reassortment events between seasonal influenza viruses were occasionally observed and reported previously in several countries [18], [19], [20]. Sometimes viral co-infection could be mistaken as reassortant virus. As a proof of concept, we have demonstrated the usefulness of this pyrosequencing method in detecting potential reassortant viruses by successful identification of the sources of all eight genes of the three vaccine production reassortant viruses IVR-148, IVR-153 and IVR-157, as well as three H1N2 viruses previously identified.

The pyrosequencing method was found to be more suitable for use with virus isolates than original specimens. It has a 100% success rate for isolates, whereas successful detection of all eight genes in original specimens depended on the quantity and possibly the quality of the specimen, pyrosequencing could only reliably detect samples with a moderate viral load, with a Ct value of less than 30 for influenza A M gene (which corresponds to about 5000 copies of matrix RNA in the reaction). Nevertheless, information on some, if not all eight gene segments from some original specimens may be sufficient for influenza A virus subtyping, as real-time RT-PCR method only relies on one or two genes for subtyping.

Apart from detecting reassortment, this method could also be applied to determine the subtype of viruses. Real-time RT-PCR is currently the most widely used assay for influenza virus typing and subtyping. However, occasionally it can fail when samples have mismatches in either the primer or probe binding regions. The pyrosequencing primers we designed here target the highly conserved regions of human influenza A viruses, therefore they may have a higher chance of detecting all subtypes of human influenza A. By detecting all eight genes, rather than targeting one or two genes as with real-time RT-PCR, pyrosequencing is more likely to detect virus subtypes accurately. A number of human influenza A viruses which could not be subtyped by real-time RT-PCR were successfully identified as H1N1pdm viruses by our pyrosequencing assay.

Using this method, we demonstrated that we could clearly distinguish between the human seasonal H1N1, H3N2 and H1N1pdm viruses that circulated in both the northern and southern hemisphere in 2008, 2009 and 2010. Moreover, the assay was able to differentiate between the two H3N2 lineages (A/Brisbane/10/2007 and A/Perth/16/2009) based on H3 HA sequence, the H275 oseltamivir-sensitive wild-type and H275Y oseltamivir-resistant mutant viruses based on N1 NA sequences, and the S31 amantadine-sensitive wild-type and N31 amantadine-resistant mutant viruses based on M2 sequence. These primers were also tested on several influenza vaccine strains which were reassortants and were able to correctly identify them as reassortants (Table 2). This method extends the more conventional use of pyrosequencing in influenza surveillance where it is mainly used for the identification of molecular markers of resistance to antiviral drugs [11], [12], [13], [14], [15].

**Table 2:** Primers for reassortment pyrosequencing.

Subtype	Gene	Forward Primer	Reverse Primer	Sequencing Primer
Seasonal and H1N1pdm	**HA**	5*TGGACWGGRATGGTAGATG	TGAACTCTTTRCCYACTGCTGTGAAC	AATGGCATTYTGTGTGCT
Seasonal H1N1	**NA**	5*ACAATGGRGCWGTGGCTG	TGCCAGTTRTCCCTGCAYACACA	TAACAGGAGCATTCCTCATA
H1N1pdm	**NA**	5*GACAGGCCTCATACAAGATCTTC	TGCCAGTTRTCCCTGCAYACACA	TAACAGGAGCATTCCTCATA
H3N2	**HA**	ACTCAAAAYGGAACAAGCTC	5*TGTGATTCTTCCTRATGCTTG	CTGCTTGCAWAAGGARATCT
H3N2	**NA**	ACTGATGGGAGTGCKTCA	5*TCCTGAACACACATAWCTGG	TTGTCAGGARGTGCTCAG
Human Influenza A	**PB2**	GTGTCAGCAGAYCCAYTAGCATC	5*TTGCCYGTDAGCACYTCTTC	ATGTGCCAYAGCAC
	**PB1**	GAYGCYGTTGCAACWACAC	5*ATGGTGGAACAGATCTTCATGATCTC	GTAYCAGRGTGCTGCAA
	**PA**	CCTTTCGTCAGTCCGAAAGAGG	5*TTGCCCTCAATGYAGCCGTTC	ACAATTGAAGAAARATTTGA
	**MP**	5* TTGGGACTCATCCTAGCTC	ACTCCTTCCGTAGAAGGSCCTCT	GTGCAMRATCCCAAT
	**NS**	GATGCCCCMTTCCTTGATCG	5*GGCATKAGCATGAACCAG	GAGRCACTTARAATGAC
	**NP**	5*CGGAATGGATCCCAGRAATGTG	ATCTCAGYRTTTCCTGGGT	GCACATTCTYTCATAAGCA

5*: indicates the primer is biotinylated at the 5′ end.

This pyrosequencing assay also has the potential to identify new influenza viruses, since the primers were designed in the highly conserved regions across different human influenza A subtypes. Although the primers were primarily designed for human influenza A viruses, some of the internal gene primers, especially MP and NS primers could also amplify most of the avian influenza A subtypes based on bioinformatics analysis, therefore at least one or more genes from some influenza A viruses with an avian origin could be successfully amplified and sequenced into unknown regions by de novo sequencing of at least 30–40 bases, which could provide enough information to identify new influenza viruses using a BLAST analysis. Even if a gene segment from a disparate animal source was encountered, a negative result would flag an unusual outcome and the whole gene segment could be further sequenced using conventional Sanger sequencing to type the reassortant.

In conclusion, for the first time we report a high-throughput pyrosequencing method for rapid identification of potential influenza A reassortant viruses derived from currently circulating subtypes. This method could also be further adapted to detect genes originating from other subtypes, such as avian influenza A subtypes H5N1, H3N8, etc by modifying some of the primers. This method is capable of facilitating the rapid identification of any new emerging reassortant influenza viruses in the future.

## Materials and Methods

### Clinical samples and virus isolates

Clinical specimens and virus isolates from Oceania and Asia were received at the WHO Collaborating Centre for Reference and Research on Influenza, Melbourne, Australia, as part of the WHO Global Influenza Surveillance Network. Viruses were isolated in Madin-Darby canine kidney (MDCK) cells (American Type Culture Collection (CCL-34)) using maintenance media (DMEM Coon's Basal Media containing sodium bicarbonate (3%) with the addition of 2 mM glutamine, 1% non-essential amino acids, 0.05% NaHCO_3_, 0.02 M HEPES, 4% penicillin and streptomycin, 2 µg/ml amphotericin B and 4 µg/ml trypsin (CSL Biotherapies)).

### RNA extraction and real-time RT-PCR

Viral RNA was extracted from clinical specimens or MDCK cell culture supernatants using either the QIAmp Viral RNA Mini Kit (QIAGEN) or the MagnaPure extraction system (Roche) according to the manufacturer's protocols. Real-time RT-PCR was performed on original clinical specimens to determine the type and subtype of the viruses using Superscript III one-step qRT-PCR kit (Invitrogen, Carlsbad, CA) with specific primers and probes [21] on an ABI 7500Fast instrument (Applied Biosystems, Foster City, CA).

### RT-PCR and pyrosequencing

cDNA was first produced from viral RNA with universal influenza RT primers (Hoffman paper ref) using ThermoScript™ RT-PCR System for First-Strand cDNA Synthesis (Invitrogen), then PCR amplification was carried out using high fidelity Platinum Taq DNA polymerase (Invitrogen) with 10 sets of primers in which either the forward or reverse primer was biotinylated (Geneworks, Adelaide, Australia) to amplify all eight genes for pyrosequencing (Table 2). PCR was done in a 50 µl reaction volume as follows: 50°C 30 min, 94°C 2 min, then 40 cycles of 94°C 30 s, 55°C 30 s, 68°C 30 sec, then a final extension at 68°C for 1 min. Single-stranded biotinylated DNA from the RT-PCR product was generated and purified using the PyroMark Vacuum Prep Workstation (Qiagen, Valencia, CA) according to the manufacturer's recommendation. Briefly, biotinylated PCR amplicons were bound to streptavidin-coated beads, and were then subjected to denaturation and washing to generate single stranded DNAs, which were then released to a PSQ plate prefilled with 40 µl annealing buffer containing pyrosequencing primer (100 µM). The sample plate was heated at 80°C for 2 min and cooled to room temperature for primer annealing. Pyrosequencing reactions were performed with the PyroMark Gold enzyme, substrate and nucleotides using the PyroMark ID instrument (Qiagen). Pyrosequencing data were analysed using Identifier software (Qiagen) with a library constructed containing the signature sequences of each gene from all subtypes to determine the origin of each gene.

### Sanger sequencing confirmation

RT-PCR products of all eight genes were amplified using specific primers. Sequencing reactions of the PCR products were carried out using BigDye Terminator (Applied Biosystems) on a 3500×L Genetic Analyzer (Applied Biosystems) according to the manufacturer's recommendation. Sequences were analysed with DNAStar Lasergene package v8.1.5.
